# The value of the erect abdominal radiograph for the diagnosis of mechanical bowel obstruction and paralytic ileus in adults presenting with acute abdominal pain

**DOI:** 10.1002/jmrs.299

**Published:** 2018-07-23

**Authors:** Wendy Z. M. Geng, Michael Fuller, Brooke Osborne, Kerry Thoirs

**Affiliations:** ^1^ International Centre for Allied Health Evidence University of South Australia Adelaide South Australia Australia

**Keywords:** Abdomen, acute, diagnostic x‐ray, ileus, intestinal obstruction, sensitivity and specificity

## Abstract

**Introduction:**

There is discord on the value of the erect abdominal radiograph for diagnosing acute abdominal pathologies. The erect radiograph can be uncomfortable for patients in pain and increases patient radiation dose.

**Aim:**

To determine if including the erect abdominal radiograph in plain abdominal radiography (PAR) improved diagnostic accuracy for identifying mechanical bowel obstruction and/or paralytic ileus in adults presenting with acute abdominal pain.

**Methods:**

PAR of 40 consecutive adults presenting with suspected bowel obstruction or paralytic ileus was retrospectively sampled and independently reviewed by two emergency department (ED) consultants and two radiology consultants for bowel obstruction and paralytic ileus across two sessions. In session 1, the assessors assessed the supine abdominal radiographs (PAR 1) and clinical details in a randomised order, and session 2, at least 6 weeks later, they assessed the supine and erect radiographs (PAR 2) and clinical details of the randomly re‐ordered cases. Computed tomography was the reference standard. Pair‐wise comparisons of receiver operating characteristic curves were calculated to assess for significant differences in participants’ diagnostic accuracy using MedCalc 16.4.3.

**Results:**

Average sensitivity, specificity and area under the receiver operating characteristic curves (AUROC) were 69.7%, 61.0% and 0.642 for PAR 1, respectively, and 80.0%, 53.4% and 0.632 for PAR 2 respectively. For AUROC there were no significant differences (*P* > 0.05) between PAR 1 and PAR 2. Intra‐rater and inter‐rater agreement improved in PAR 2.

**Conclusion:**

There was no statistically significant improvement in diagnostic accuracy when including the erect radiograph in PAR for the acute abdomen.

## Introduction

Plain abdominal radiography (PAR) is often the initial diagnostic imaging tool for patients presenting with acute abdominal pain.^1^ PAR in the acute setting may consist of supine and erect abdominal radiographs and an erect chest radiograph.^2^, ^3^ The erect chest view is recommended for diagnosing chest pathologies, such as pneumonia, that mimic the symptoms of an acute abdomen.^4^ The exposure parameters of the chest x‐ray also give greater visualisation of free gas under the diaphragm when a hollow visceral perforation is suspected.^5^ The erect abdominal radiograph (EAR) may not be necessary because most findings are demonstrated on the supine abdominal radiograph (SAR).^2^, ^3^, ^6^, ^7^, ^8^ However, air–fluid levels are only seen on the EAR and are a significant radiological sign for diagnosing acute small bowel obstruction.^9^, ^10^ Bowel obstruction is one of the most common diagnoses in patients presenting with acute abdominal pain, accounting for 12.6–21.8% of emergency admissions.^11^, ^12^, ^13^ This condition prevents the distal flow of intestinal contents, and can be of a mechanical or functional nature. Mechanical bowel obstruction occurs when a physical barrier prevents intestinal flow, whereas paralytic ileus is a functional impairment of the bowel wall or nervous system.^14^ Immediate life‐saving surgical intervention is required in some cases of bowel obstruction.^15^ Therefore, a rapid diagnosis is required for these conditions. It would be helpful for radiographers to know the value of the EAR, as it can be a difficult radiograph for both the radiographer and the patient to achieve when the patient is in pain or disabled and adds to patient radiation dose. There has also been limited investigation into the diagnostic value of the EAR, with most studies undertaken over two decades ago and without using a standardised reference standard.^2^, ^7^, ^16^, ^17^, ^18^, ^19^, ^20^, ^21^

The diagnostic value of the EAR may vary depending upon the experience of the interpreting doctor. Doctors with less experience may find it difficult to interpret abdominal radiographs that appear to have normal anatomy but have an unusual bowel gas pattern.^22^ The addition of an EAR or decubitus abdominal radiograph may be helpful to these doctors as it provides more information or, as others argue, it may provide misleading information.^17^, ^23^

We undertook this study to (1) determine if the inclusion of the EAR in PAR improves diagnostic accuracy in identifying mechanical bowel obstruction and/or paralytic ileus in adults presenting with acute abdominal pain and (2) to determine if there is a difference in the interpretations of abdominal radiographs between doctors.

## Methods

This study was undertaken at Flinders Medical Centre, South Australia. Ethical approval was granted by the Southern Adelaide Clinical Human Research Ethics Committee and the Human Research Ethics Committee, University of South Australia.

### Study design

The diagnostic accuracy of two different plain abdominal radiography (PAR) protocols was compared by retrospectively reviewing patient cases who had presented with acute abdominal pain and were clinically suspected of mechanical bowel obstruction or paralytic ileus.

### Case selection

Cases were retrospectively selected from the picture archive and communication system (PACS) and radiology information system (RIS) of Flinders Medical Centre. Consecutive patient cases from 29 November 2016 were sampled in a backwards time direction until 40 cases were retrieved. Case inclusion criteria were that the patient was 18–65 years old, presented to the emergency department (ED) with acute abdominal pain and clinical suspicion of mechanical bowel obstruction or paralytic ileus, have undergone SAR and EAR within 24 h of presentation to the ED and undertaken an abdominal computed tomography (CT) scan within 4 h after PAR. Cases were excluded if the patient was institutionalised, had psychiatric or neurological disorders or had an equivocal CT result. Our sample size of 40 cases was based on an estimation that 20 of the 40 cases would have mechanical bowel obstruction or paralytic ileus. According to a published table^24^ this would provide an 80% probability of detecting a 20% difference in diagnostic accuracy (*P* < 0.05).

### Index tests

Two PAR protocols were compared for each case. PAR 1 consisted of only SAR, and PAR 2 consisted of SAR and EAR.

Two radiology consultants and two ED consultants, all of whom were experienced in interpreting PAR in the acute setting, participated in this study. Post‐registration experience ranged from 25 to 27 years, and 10 to 13 years for the radiologists and ED consultants respectively. Each assessor independently assessed the two PAR protocols, with a minimum 6‐week interval between starting PAR 2 and completing PAR 1. Clinical information from the request form for each case was included with the images.

**Figure 1:**
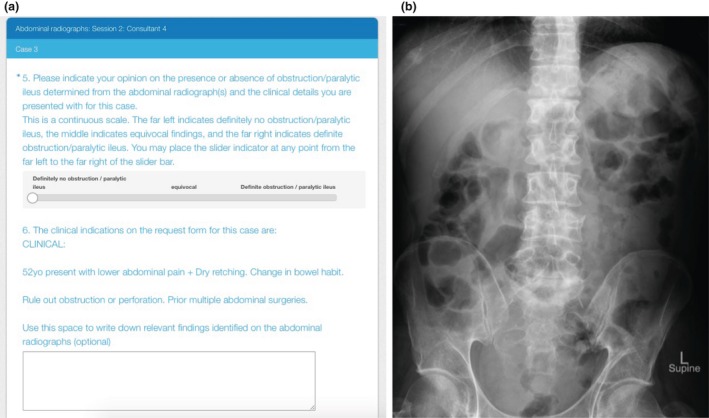
(a) An example of a patient case presented on the online survey. (b) A patient's supine abdominal x‐ray presented on the survey.

Cases were presented to the assessors in a randomised order using an electronic survey tool (SurveyMonkey Inc., California, USA) (Fig. 1). The assessors could only view the series in a forward direction to limit comparisons with previous cases. The assessors were instructed on how to indicate their diagnostic assessment using the visual analogue scale (VAS) in the survey. This consisted of a 0–100 continuous scale which provided an indication of how sure each participant was of their assessment. The left and right ends of the scale were labelled with ‘definitely no obstruction/paralytic ileus’ and ‘definite obstruction/paralytic ileus’ respectively. The assessors could undertake the assessments at their convenience and complete the survey across multiple sittings.

### Reference standard

The radiology report for the CT scan from each case was used to categorise each case as ‘positive’ or ‘negative’ and served as the reference standard. The assessors were blinded to the CT results. Each CT scan was reported as per the department protocol, with an available consultant radiologist or radiology registrar producing the report. All reports produced by registrars were checked by consultants with an addendum report issued if necessary. CT has a reported sensitivity, specificity and accuracy of 90–94%, 93–100% and 94–95%, respectively, for the detection of mechanical bowel obstruction,^25^, ^26^, ^27^, ^28^ and has the highest accuracy for the differential diagnosis of mechanical SBO and post‐operative paralytic ileus.^29^

### Statistical analysis

All statistical calculations were performed using MedCalc 16.4.3 (MedCalc Software, Ostend, Belgium). Two receiver operating characteristic (ROC) curves were generated for each assessor using continuous data from the VAS scale (index test) and binomial data from the CT radiology report (reference test). The first ROC for each participant represented their diagnostic assessments when using only the supine abdominal radiograph (PAR 1). The second ROC represented their diagnostic assessments when using both the supine and erect abdominal radiograph (PAR 2). Area under the receiver operating characteristic curves (AUROC) were calculated for each participant and pair‐wise comparisons (*P* < 0.05) were made between PAR protocols and assessors using the statistical method of Delong, Delong and Clarke‐Pearson.^30^ An AUROC of ‘0’ indicates that the diagnostic test is consistently incorrect at differentiating diseased from non‐diseased states, ‘1’ indicates the test to be always correct and ‘0.5’ indicates a chance level of differentiation.^31^ The Youden's index was calculated to determine the optimal threshold point (criterion value), and its associated sensitivity and specificity.^32^, ^33^

### Agreement testing

Ten duplicate cases for each PAR protocol were used to test for intra‐rater agreement of each assessor's diagnostic interpretations. Each assessor was given different duplicate cases randomly mixed into the case series. Intra‐class correlation coefficients (ICC) were calculated using a two‐way mixed‐effects model based on a single measure and absolute agreement. Inter‐rater agreement was tested by comparing diagnostic assessments between the assessors for each PAR. Agreement was tested by calculating the ICC using a two‐way mixed‐effects model based on the average of two raters and absolute agreement.^34^ An ICC of less than 0.5, between 0.5 and 0.75, between 0.75 and 0.9 and more than 0.9 demonstrates poor, moderate, good and excellent agreement respectively.^34^

## Results

The 40 cases included 17 females and 23 males (mean age 49.0 ± 9.42 years). Table 1 demonstrates clinical presentations of all cases. Fifteen (38%) cases had bowel obstruction or paralytic ileus diagnosed by CT. The average time between patient admission and PAR was 194±155 min and between PAR and CT was 137±60.5 min. The time interval between the two testing sessions was 7–8 weeks for the ED doctors, 6 weeks for one radiologist and 10 weeks for the second radiologist.

**Table 1:** Clinical symptoms and computed tomography diagnosis

Clinical symptoms (*n*)	Computed tomography diagnosis (*n*)
Abdominal pain (31)	Bowel obstruction (13)
? bowel obstruction/ileus (38)	Paralytic ileus (2)
Abdominal distension (9)	Appendicitis (6)
Decrease/no flatus or bowel not open (13)	Inflammation of the bowel (2)
Nausea/vomiting (15)	Perforation (2)
? perforation (16)	Hernia (3)
Known hernia (4)	Other abnormalities (8)
? hernia (2)	No intra‐abdominal abnormality (4)
Other clinical details indicative of bowel obstruction/ileus (13)	

### Diagnostic accuracy of consultants’ interpretations

Diagnostic accuracy data for each assessor for PAR 1 and PAR 2 are presented in Table 2. Across all assessors, the AUROC ranged from 0.581 to 0.712 with an average of 0.632 for PAR 1 and from 0.565 to 0.673 with an average of 0.632 for PAR 2. There were no significant differences (*P* > 0.05) in AUROC between the two PAR protocols. Average sensitivity and specificity were 69.7% and 61.0% for PAR 1, respectively, and 80.0% and 53.4% for PAR 2 respectively (Table 3). There was a wide variation in optimum criterion values, sensitivity and specificity values between assessors and between PAR protocols.

**Table 2:** Diagnostic accuracy of assessments made by each doctor for each protocol

Assessor	AUROC (95% CI)		Difference in AUROC (95% CI)	Significant level (*P*‐value)
	PAR 1	PAR 2		
1 (ED)^1^	0.642 (0.472 to 0.788)	0.565 (0.397 to 0.723)	0.0764 (−0.0904 to 0.243)	0.370
2 (ED)	0.581 (0.415 to 0.735)	0.673 (0.507 to 0.813)	0.0920 (−0.0637 to 0.248)	0.247
3 (radiology)	0.712 (0.547 to 0.844)	0.651 (0.484 to 0.794)	0.0613 (−0.112 to 0.235)	0.489
4 (radiology)^2^	0.634 (0.465 to 0.782)	0.637 (0.468 to 0.785)	0.00286 (−0.212 to 0.218)	0.979

ED, emergency department; >, greater than; %, percentage; CI, confidence interval; AUROC, area under the receiver operating characteristic curve.

^1^Case 1 in PAR 2 removed from statistical analysis due to data management error.

^2^Case 13 in PAR 1 removed from statistical analysis due to data management error.

**Table 3:** Sensitivity and specificity at criterion values

Assessor	Criterion value^1^		Sensitivity (%) (95% CI)		Specificity (%) (95% CI)	
	PAR1	PAR2	PAR1	PAR2	PAR1	PAR2
1 (ED)^2^	>52	>26	73.3 (44.9–92.2)	86.7 (59.5–98.3)	56.0 (34.9–75.6)	37.5 (18.8–59.4)
2 (ED)	>38	>51	86.7 (59.5–98.3)	73.3 (44.9–92.2)	36.0 (18.0–57.5)	56.0 (34.9–75.6)
3 (radiology)	>86	>81	40.0 (16.3–67.7)	73.3 (44.9–92.2)	96.0 (79.6–99.9)	68.0 (46.5–85.1)
4 (radiology)^3^	>29	>8	78.6 (49.2–95.3)	86.7 (59.5–98.3)	56.0 (34.9–75.6)	52.0 (31.3–72.2)

ED, emergency department; >, greater than; %, percentage; CI, confidence interval.

^1^Criterion value is the value on the receiver operating characteristic curve where sensitivity and specificity – 1 is maximum.

^2^Case 1 in PAR 2 removed from statistical analysis due to data management error.

^3^Case 13 in PAR 1 removed from statistical analysis due to data management error.

### Intra‐rater agreement

Moderate‐to‐excellent intra‐rater agreement (ICC of 0.551–0.939) was achieved for PAR 1 (Table 4). Adding EAR to PAR 2 increased the intra‐rater agreement of diagnostic interpretations for all assessors except one radiology consultant.

**Table 4:** Participants’ agreement in sessions 1 and 2

Assessor(s)	ICC for PAR 1 (95% CI)	ICC for PAR 2 (95% CI)
1 (ED)	0.676 (0.144 to 0.907)	0.993 (0.976 to 0.998)^1^
2 (ED)	0.551 (−0.0964 to 0.867)	0.794 (0.363 to 0.945)
3 (radiology)	0.774 (0.307 to 0.939)	0.976 (0.906 to 0.994)
4 (radiology)	0.939 (0.787 to 0.984)	0.667 (0.142 to 0.903)
Radiology consultants (combined)	0.630 (0.288 to 0.807)^2^	0.617 (0.0844 to 0.823)
ED consultants (combined)	0.413 (−0.116 to 0.690)	0.859 (0.579 to 0.940)^2^
All consultants (combined)	0.733 (0.558 to 0.846)^2^	0.8650 (0.7534 to 0.9275)^2^

ICC, intra‐class correlation coefficient; %, percentage; CI, confidence interval; ED, emergency department.

^1^11 duplicate cases analysed for intra‐rater agreement.

^2^39 duplicate cases analysed for intra‐rater agreement.

### Inter‐rater agreement

Moderate‐to‐good agreement (ICC of 0.413–0.733) between the assessors was achieved for PAR 1, and good‐to‐excellent agreement was achieved for PAR 2 (Table 4).

## Discussion

Both PAR protocols demonstrated low‐to‐moderate diagnostic accuracy for identifying mechanical bowel obstruction and/or paralytic ileus in adults presenting with acute abdominal pain. We found no significant differences in the overall accuracy between the two protocols. This is consistent with other studies which have demonstrated limited value of the EAR.^16^, ^19^, ^21^

We found no significant differences in overall diagnostic accuracy between the assessors. This is in contrast to the study by Thompson et al.,^10^ which found more senior and experienced radiologists to be more accurate and confident in diagnosing SBO using PAR than radiologists with less than 5 years experience. The assessors in our study all had over 10 years of experience suggesting that the effect of experience on learning diminishes after 10 years for both radiologists and ED doctors. Other authors have compared the interpretations made by radiologists and non‐radiologists, finding that non‐radiology doctors mostly missed, misinterpreted or identified irrelevant radiological features.^7^, ^17^ Improved image interpretation training for non‐radiologic doctors since the 1980s is a potential reason for the discrepancy in our findings and these earlier studies.

Intra‐rater and inter‐rater agreement increased when the EAR radiograph was added to the protocol. This improvement was most profound for inter‐rater agreement between the two ED consultants, which more than doubled when the EAR radiograph was added. Factors for this result may include both the doctors’ speciality or years of experience which was different from the radiologists. However, the wide confidence interval for some results indicates that the 10 duplicates cases used to test reliability may not have been enough to give a true indication of reliability.

We asked the assessors to rate, on a continuous scale, the definite presence or absence of the conditions rather than to dichotomise their assessment into ‘positive’ or ‘negative’. This reflects radiologic practice, where descriptors such as ‘probable’, ‘unlikely’ or ‘apparent’ are commonly used.^35^ There were wide variations in the sensitivity and specificity values between the assessors. This variation was also demonstrated in previous studies reporting wide ranges of sensitivity (19–96.2%) and specificity (57–100%) for diagnosing SBO.^10^, ^36^, ^37^, ^38^, ^39^, ^40^ Our sensitivity and specificity values for SAR (40–86.7%) and SAR combined with EAR (73.3–86.7%) were lower than that reported by Tie and Edwin,^21^ who reported 88.5% and 92.5% sensitivity for SAR and SAR combined with EAR respectively. These differences may be accounted for by disease prevalence, which was higher in our study (37.5%), compared to 11.6% reported by Tie and Edwin.^21^, ^41^

Our results do not strongly support the inclusion of EAR in PAR, with the likelihood of additional confirmatory imaging such as CT still being required. This study builds on the existing limited body of evidence investigating the value of the EAR when bowel obstruction or paralytic ileus is suspected. We used CT as a consistent and sole reference standard. Compared to other studies where clinical history was not revealed to interpreting doctors, the assessors in our study reviewed clinical details together with the radiograph, reflecting normal practice.^7^, ^16^, ^17^, ^21^ We did not seek to identify radiographic signs of bowel obstruction and paralytic ileus.

PAR is still used in many practices as the initial imaging modality for patients experiencing acute abdominal pain due to its low cost and wide availability.^10^, ^21^, ^42^ Based on a wide range of diagnostic values in previous studies, and the low diagnostic accuracy and variations in sensitivity and specificity across assessors reported in this study, patients with a negative or positive PAR are still likely to undergo another confirmatory test such as CT. Thus, the use of PAR for patients presenting with an acute abdomen should be reviewed. Rather than investing in more rigorous prospective studies with larger sample sizes in PAR, perhaps consideration should be given to studying the feasibility of other diagnostic tools such as low‐dose CT (LDCT) in place of PAR. LDCT has been shown to give significantly higher diagnostic yield than PAR for adults with acute abdominal pain and can potentially reduce the number of further imaging investigations with almost equal or only slightly higher radiation dose.^43^, ^44^, ^45^

## Limitations

The retrospective study design and sampling methods are limitations. The criteria of including patients who had both PAR and CT may have created bias to the sample to include more cases referred for CT investigation due to equivocal abdominal radiographs, and more cases with unequivocal CT findings. Patients who were institutionalised, or who had psychiatric or neurological disorders were also excluded and therefore results cannot be applied to these patient groups. Confidence intervals for many of our outcome measurements were wide, raising the possibility that the sample was not large enough to detect true significant differences.

Alternative radiographs to the EAR, such as decubitus abdominal or erect chest radiographs, were not considered in this study. This study used CT as the sole and standardised reference standard due to its high accuracy, however, it is not 100% accurate for diagnosing bowel obstruction and paralytic ileus.^27^, ^28^

We minimised the risk of memory recall bias and cross‐referencing between assessors by randomising the order of case presentation for each assessor, and a time interval of at least 6 weeks between interpretation of each protocol.

The survey tool restricted the use of ‘windowing’ of images, making measurements which may have been used to facilitate the determination of the degree of bowel loop distension, and a standardised film reading environment. In normal practice, radiographs are viewed on high‐definition computer screens. However, the assessors’ interpretations were unlikely to have been affected as radiologic signs for bowel obstruction and paralytic ileus do not need high resolution.^46^ Another potential limitation is that the departmental CT reporting process did not control for intra‐reader and inter‐reader variability between different radiology consultants.

## Conclusion

Both PAR protocols demonstrated low diagnostic accuracy for the identification of mechanical bowel obstruction and paralytic ileus in adults presenting with acute abdominal pain raising questions about the value of PAR in this setting. The addition of the EAR to the SAR gave a slight but insignificant increase in diagnostic accuracy, and improved the intra‐rater and inter‐rater agreement, particularly for ED consultants. Radiographers performing PAR in the investigation of mechanical bowel obstruction and paralytic ileus should be aware of the limited value of the erect radiograph, especially in situations where it is technically difficult to achieve, patient tolerance is low and the radiographs are to be viewed by an experienced consultant radiologist.

## Conflict of Interest

The authors declare no conflict of interest.
